# Surveillance Donor-derived Cell-free DNA Allows for the Safe Reduction in Protocol Transbronchial Biopsies After Lung Transplantation

**DOI:** 10.1097/TXD.0000000000001901

**Published:** 2026-01-12

**Authors:** Kashika Goyal, David J. Ross, Bronwyn Small, Namita Trikannad, Sangeeta Bhorade, Justin P. Rosenheck

**Affiliations:** 1 Department of Internal Medicine, The Ohio State University, Wexner Medical Center, Columbus, OH.; 2 Department of Medical Affairs, Natera, Inc., Austin, TX.

## Abstract

**Background.:**

Protocol transbronchial biopsies (TBBx) are standard of practice (SOP) in most lung transplantation (LT) centers for surveillance of acute cellular rejection. In the context of higher LT center volumes, our program experienced increasing difficulty scheduling SOP protocol TBBx at months 1, 3, 6, 9, and 12 post-LT. As part of a Quality Assurance Performance Improvement initiative, we designed this prospective study to assess the efficacy and safety of adopting donor-derived cell-free DNA (dd-cfDNA) surveillance in lieu of protocol TBBx during the initial year after LT at our center.

**Methods.:**

Enrolled LT patients with “low-risk” dd-cfDNA (<1.0%) had their 9-mo (9M) protocol TBBx omitted, whereas the 9M protocol TBBx was performed when dd-cfDNA indicated a “high-risk” result (≥1.0%) or for clinical indications. All patients received a protocol TBBx at 12M to assure detection of subclinical acute cellular rejection. We also assessed clinical data from the first year, including change in forced expiratory volume-1 s, donor-specific antibodies, and performed a health economic analysis.

**Results.:**

Among 78 enrolled LT patients, 24 were “high risk” (8 with omitted 9M protocol TBBx because of clinical contraindications), and 54 “low risk” (41 with omitted 9M protocol TBBx) by dd-cfDNA. Of the patients with omitted 9M protocol TBBx, 10.2% (5/49) showed rejection at 12 mo compared with 20.7% (6/29), at either the 9M or 12M protocol TBBx. Median change in forced expiratory volume-1 s (baseline—12M) was similar between “high-risk” and “low-risk” cohorts (*P* = 0.592) and for “omitted” versus “performed” 9M protocol TBBx cohorts (*P* = 0.271).

**Conclusions.:**

Our Quality Assurance Performance Improvement study offered assurances for safety, efficacy, and reduction in healthcare costs when implementing SOP dd-cfDNA surveillance, with a >75% reduction in 9M protocol TBBx procedures among patients who were “low risk” by dd-cfDNA.

## INTRODUCTION

Long-term outcomes after lung transplantation (LT) remain suboptimal with survival rates of 88.5% at 1 y, 71.3% at 3 y, 59.7% at 5 y, and 31.8% at 10 y after LT.^1^ Acute rejection (AR), which can occur in >50% of patients within the initial year posttransplant, is a significant risk factor for the development of chronic lung allograft dysfunction and graft loss.^2^ Although protocol transbronchial biopsies remain the standard of practice (SOP) in a majority of US centers, a recent meta-analysis had found no difference in detection of AR when analyzing per protocol (surveillance), when compared with clinically-indicated transbronchial biopsy (TBBx) procedures.^3^ However, the lack of robust data from an appropriately powered, randomized, and controlled clinical study has deterred large-scale modification of the historical SOP, such as a shift to surveillance with a noninvasive plasma biomarker, such as donor-derived cell-free DNA (dd-cfDNA).^3,4^

Our study aimed to analyze both efficacy and safety when adopting dd-cfDNA surveillance in lieu of protocol TBBx during the initial year after LT at our center. This analysis was prompted by a Quality Assurance and Performance Improvement (QAPI) Initiative to address the challenge of scheduling increased TBBx demand because of higher LT center volume according to our center’s SOP at months 1, 3, 6, 9, and 12 posttransplant.^5^ In our QAPI protocol, we specifically targeted the 9-mo (9M) protocol TBBx for potential omission among patients found to be low risk by dd-cfDNA, a clinically validated biomarker for the detection of AR.^6-8^ The 12-mo protocol TBBx was maintained to provide reassurance in the event of an asymptomatic “subclinical” AR event that was not detected at 9M.

## MATERIALS AND METHODS

### Study Overview

All patients in this study received basiliximab induction and were maintained on triple therapy (tacrolimus, mycophenolate mofetil, and prednisone). In the first-year posttransplant, all patients received at least monthly spirometry and were seen in the clinic monthly. Our center has routinely performed protocol TBBx as SOP at 1, 3, 6, 9, and 12-mo posttransplant. Patients with A0 and asymptomatic A1 acute cellular rejection (ACR) are typically not treated with augmented immunosuppression, whereas symptomatic A1 and all A2+ patients are treated.

**FIGURE 1.**
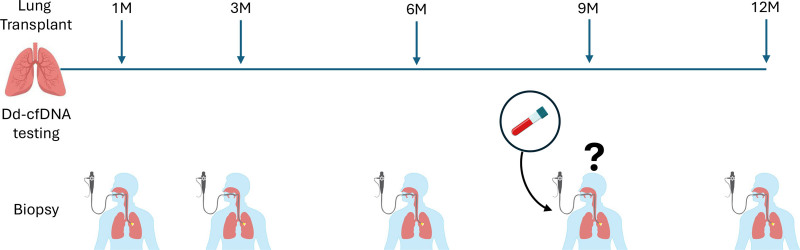
Study protocol schematic—the clinical decision to schedule or omit the 9M bronchoscopy and TBBx procedure was based on the dd-cfDNA test results (dd-cfDNA < 1.0% or “low risk” vs dd-cfDNA ≥ 1.0% or “high risk”) performed at the 8-mo time point. dd-cfDNA, donor-derived cell-free DNA; TBBx, transbronchial biopsy.

We designed this prospective study as part of a QAPI initiative,^5^ in which the 9M protocol TBBx was omitted in clinically stable patients deemed at “low risk” by dd-cfDNA (<1%); the 12M protocol TBBx was completed for all patients (Figure 1). The study was considered IRB-exempt by The OSU Human Subjects Committee. Dd-cfDNA testing (the Prospera^TM^ Lung test; Natera, Inc., San Carlos, CA) was performed at 8M, approximately 4 wk before the 9M timepoint, to assign a risk profile. If dd-cfDNA and clinical status were reassuring, their 9M protocol TBBx was canceled. Routine clinical data included chest radiography, in-laboratory spirometry, and laboratory and immunogenetic testing for HLA class I and class II donor-specific antibodies (DSAs). Flexible bronchoscopy TBBx and bronchoalveolar lavage were performed in accordance with International Society for Heart & Lung Transplantation (ISHLT) guidelines and the assignment of histopathologic grading.^9^ Pulmonary functional testing (PFT), specifically spirometry, was performed in accordance with guidelines established by the American Thoracic Society and European Respiratory Society.^10^ As an adaptation of the established ISHLT guidelines to determine peak forced expiratory volume-1 s (FEV_1_),^11^ we defined the “baseline” FEV_1_ as the mean of the 2 measurements at least 3 wk apart obtained immediately before the 9M visit, typically 2–3 mo preceding the 9M timepoint. We used this “baseline” as a comparator for FEV_1_ measurements taken at 9M and 12M, and we also considered the change in FEV_1_ (ΔFEV_1_) for the intervals “baseline to 9M,” “9M to 12M,” and “baseline to 12M.”

### dd-cfDNA Surveillance

The workflow for dd-cfDNA testing has been previously described.^6,12^ Briefly, blood was collected in 10-mL cell-free DNA Streck tubes. Plasma samples were analyzed using the Prospera^TM^ test at Natera’s Clinical Laboratory Improvement Amendments–certified, College of American Pathologists-accredited laboratory (San Carlos, CA). The cfDNA was amplified using a massively multiplexed polymerase chain reaction assay targeting a curated panel of >13 000 single-nucleotide polymorphisms designed to maximize variant frequency across ethnicities.^13^ For each sample, amplicons were sequenced by next-generation sequencing, performed on the Illumina NovaSeq 6000 on rapid run with an average of 14–15 million reads per sample and sequencing data processed to estimate the fraction of dd-cfDNA (expressed as a percentage) in relation to total cfDNA.

### Analysis

ACR grades on biopsies (ISHLT grade A0, A1, A2, A3, A4, AX), the development of donor-specific HLA Class I and Class II antibodies (DSA) during the 9M–12M interval and ΔFEV_1_ between “baseline” and 9M, 9M and 12M, and “baseline” and 12M were analyzed. Enrolled patients were divided into cohorts based on binary clinical criteria including 8M dd-cfDNA <1.0% (“low-risk”) versus ≥1.0% (“high-risk”), or whether the 9M transbronchial biopsy (TBBx) was “performed” versus “omitted.” Mann-Whitney U test was used for analysis of unpaired cohort comparisons of continuous variables. Box and Whisker plots illustrated cohort distributions with median, 25%–75% interquartile range (IQR), and jittered strip plots.

## RESULTS

### Demographics and Data Overview

**FIGURE 2.**
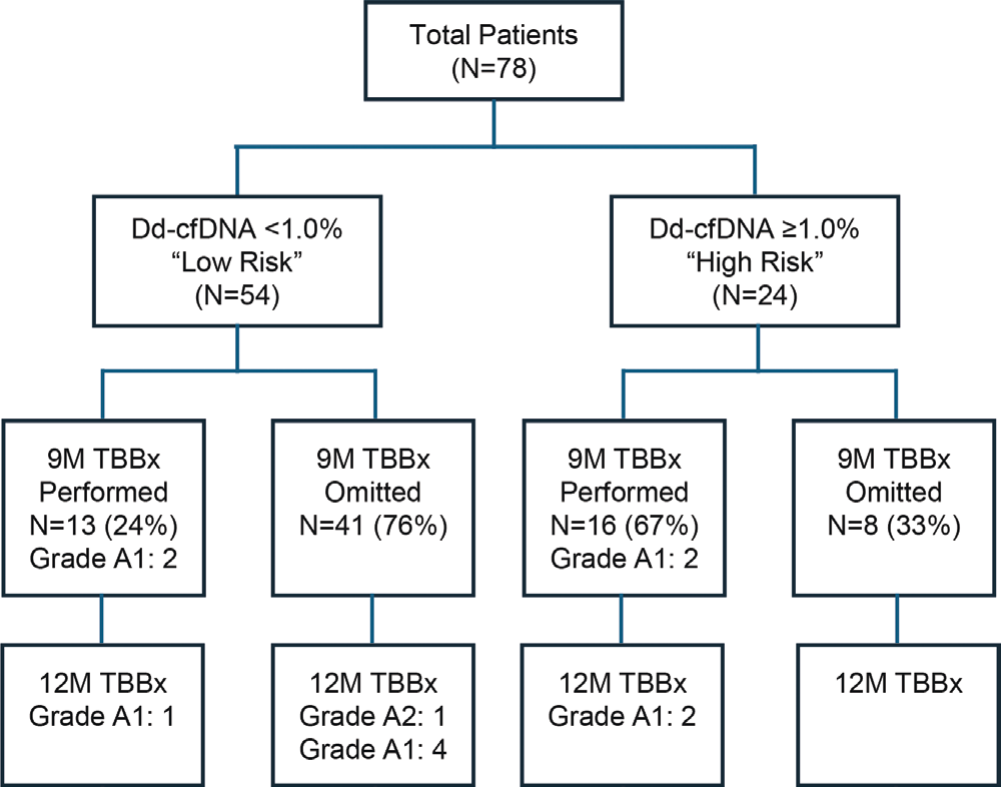
CONSORT diagram. ACR per ISHLT criteria were inclusive of grade A1 or grade A2, as shown. ACR, acute cellular rejection; ISHLT, International Society for Heart & Lung Transplantation.

A total of 78 LT patients with 12M complete data were enrolled between January 7, 2021, and January 5, 2025 (bilateral LT = 63, right single LT = 8, left single LT = 7; Figure 2). Native lung diseases for LT included idiopathic pulmonary fibrosis (N = 32), nonspecific interstitial pneumonitis (N = 7), chronic obstructive pulmonary disease (N = 15), combined pulmonary fibrosis and emphysema (N = 5), connective tissue disease–interstitial lung disease (N = 6), SARS-Co(V)-2 acute respiratory disease syndrome (N = 3), SARS-Co(V)-2 interstitial pulmonary fibrosis (N = 3), pulmonary arterial hypertension (N = 2), sarcoidosis (N = 1), retransplant (N = 1), cystic fibrosis (N = 1), bronchopulmonary dysplasia (N = 1), and hypersensitivity pneumonitis (N = 1). There were no deaths or retransplantations for graft failure among the QAPI enrolled patients.

**FIGURE 3.**
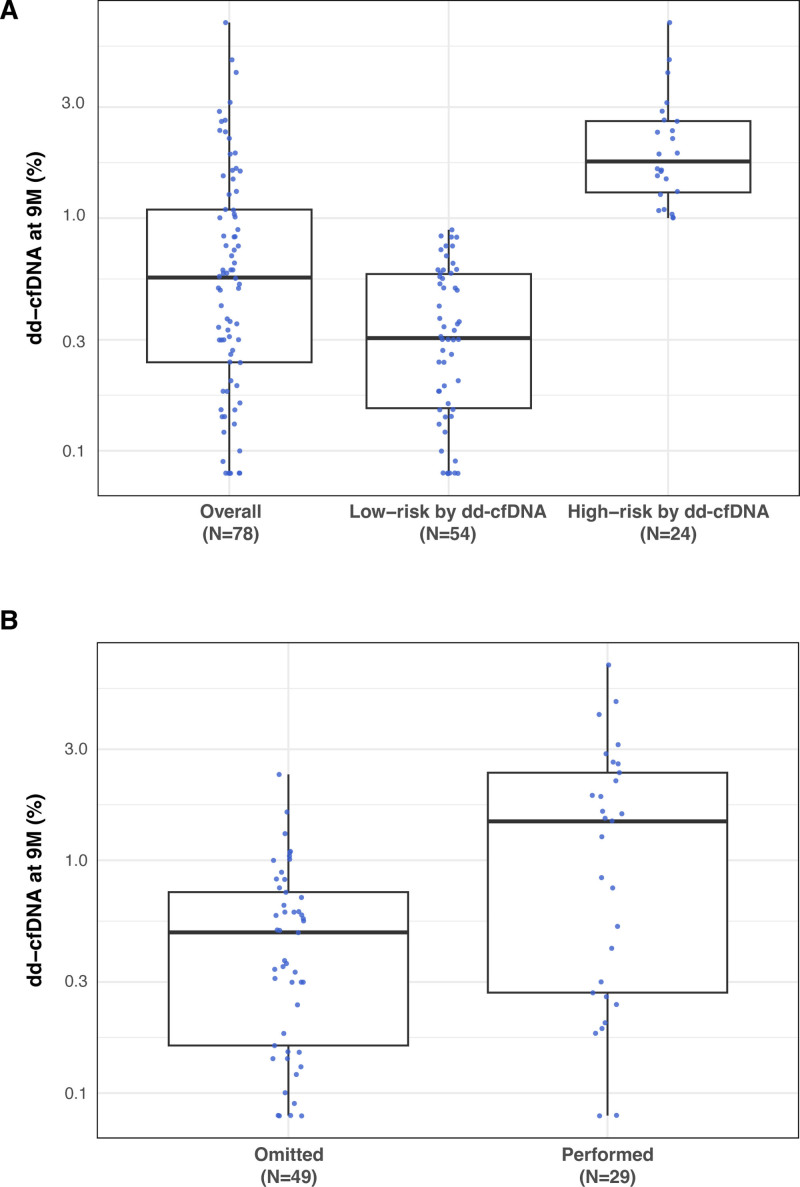
A, Distribution of dd-cfDNA (%) values for the overall, “low-risk,” and “high-risk” cohorts based on dd-cfDNA results from the 8-mo timepoint, prior to the potential 9M protocol TBBx procedure. B, Distribution of dd-cfDNA (%) values depicted for “performed” or “omitted” 9M biopsy cohorts. Box and Whisker plot – median (line), box (25%–75% IQR), Whiskers (1.5 × IQR), outlier points. dd-cfDNA, donor-derived cell-free DNA; IQR, interquartile range; TBBx, transbronchial biopsy.

In N = 49 (62.8%) total patients, the protocol TBBx procedure was omitted, whereas in N = 29 (37.2%), it was performed at the discretion of the clinician regardless of the biomarker risk assessment. The 9M TBBx was omitted in 75.9% of patients with a “low risk” dd-cfDNA assessment. In 16 of 24 (67%) patients with a “high risk” and 13 of 54 (24%) with “low-risk” 9M dd-cfDNA result, the protocol TBBx was performed for histopathologic assessment. The median time posttransplant to the dd-cfDNA assessment (N = 78 patients) was 249 (239–267) d. Median (IQR) time posttransplant to the performed 9M protocol TBBx was 280 (266–287) d. The median dd-cfDNA result of the entire cohort before 9M assessment was 0.56% (0.24–1.09) with medians of 1.76% (1.28–2.62) and 0.31% (0.15–0.58), among the “high risk” and “low risk” by the dd-cfDNA cohort, respectively (*P* < 0.0001). The dd-cfDNA distributions before the 9M TBB procedure are depicted for “high-risk” (N = 24) and “low-risk” (N = 54) patient cohorts in Figure 3A and for protocol TBBx omitted (N = 49) and performed (N = 29) cohorts in Figure 3B.

### Rejection Rates Were Lower in the Biopsy-omitted and “Low Risk” by dd-cfDNA Cohorts

Of the 54 “low risk” by dd-cfDNA patients, 13 had the 9M protocol TBBx performed; 2 were found to have minimal rejection (A1), one of which was persistent at the 12M protocol TBBx; neither of these were treated with augmented immunosuppression, and both had stable PFT throughout the evaluated period. The remaining 41 patients omitted the 9M protocol TBBx; 5 of these were later found to be rejecting at 12M (A1: n = 4, A2: n = 1). Two of these (A1: n = 1, A2: n = 1) received pulsed-dose corticosteroids, and all 5 had stable PFT during the evaluated period. Of the 24 “high-risk” by dd-cfDNA patients, 16 had their 9M protocol TBBx performed; 2 of these were found to have rejection on the 9M protocol TBBx (both A1), whereas a different 2 were found to have rejection at the 12M protocol TBBx (both A1), none of which were treated with methyl prednisolone The remaining 8 had an omitted 9M protocol TBBx; none were found to have rejection at 12M. Of the 49 patients whose 9M protocol TBBx was omitted, 5 patients (10.2%) were found to have rejection at 12 mo, according to histology. In contrast, of the 29 patients who did receive a 9M protocol TBBx, 20.7% (6/29) were found to have rejection at either the 9M or 12M protocol TBBx. Of the “low risk” by dd-cfDNA patients, 13.0% (7/54) had rejection at either the 9M or 12M biopsy, compared with 16.7% (4/24) in the “high-risk” by dd-cfDNA patients.

### There was no Difference in FEV_1_ Values at 12M or ΔFEV_1_ for the Interval of Baseline to 12M for the Omitted 9M Protocol Biopsy Cohort

**FIGURE 4.**
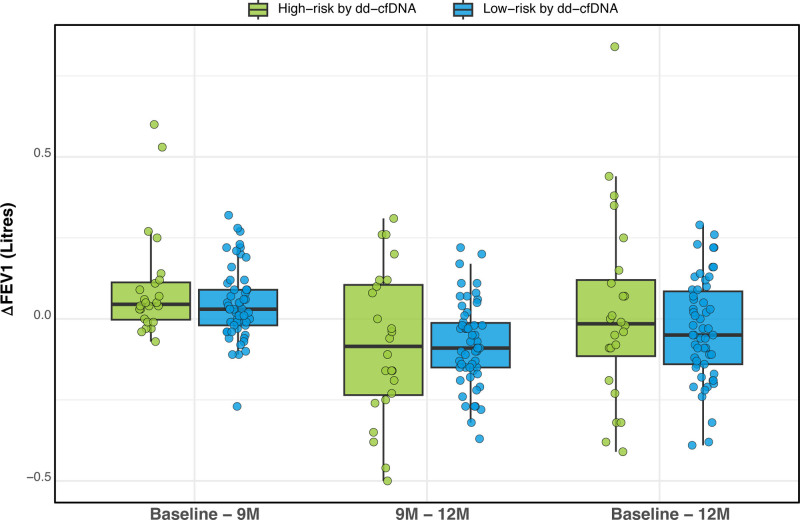
ΔFEV_1_ for time intervals of “baseline” to 9M, 9M to 12M, and baseline to 12M timepoints in the “low-risk” and “high-risk” cohorts as assessed by dd-cfDNA testing. Box and Whisker plot – median (line), box (25%–75% IQR), Whiskers (1.5 × IQR), outlier points. Mann-Whitney U test for unpaired cohort comparisons (*P* < 0.05). ΔFEV_1_, change in forced expiratory volume-1 s; dd-cfDNA, donor-derived cell-free DNA; IQR, interquartile range; TBBx, transbronchial biopsy.

The absolute FEV_1_ values at baseline, 9M and 12M, along with the changes in FEV_1_ measurements between the cohorts were compared between the high- and low-risk cohorts; no significant differences were observed (Table 1; Figure 4). In the “low-risk” cohort with 9M protocol TBBx procedure omission, the 12M TBBx subsequently demonstrated ACR in 5 of 41 patients (12.2%; ISHLT grades A1:4, A2:1). For these 5 ACR patients in the “low-risk” cohort with 9M protocol TBBx omission, the ΔFEV_1_ interval (baseline–12M) was not different when compared with those without later ACR (N = 36; −0.08 [−0.21, 0.05] L versus −0.06 [−0.15, 0.05, *P* = 0.811] L).

**TABLE 1.** Data stratification by dd-cfDNA (%) assessment of “low risk” or “high risk” before the scheduling of 9M protocol bronchoscopy and transbronchial biopsies

	Overall, N = 78	“High risk” (dd-cfDNA ≥ 1.0%), N = 24	“Low risk” (dd-cfDNA < 1.0%), N = 54	*P*
dd-cfDNA (%)	0.56 (0.24–1.09)	1.76 (1.28v2.62)	0.31 (0.15–0.58)	
FEV_1_ baseline (L)	2.04 (1.81–2.60)	2.00 (1.69–2.56)	2.07 (1.85–2.66)	0.357
FEV_1_ at 9M	2.06 (1.68–2.51)	2.02 (1.62–2.29)	2.07 (1.75–2.61)	0.304
FEV_1_ at 12M	2.12 (1.83–2.62)	2.08 (1.68–2.43)	2.13 (1.86–2.74)	0.229
ΔFEV_1_ (baseline–9M)	0.04 (−0.01 to 0.11)	0.05 (−0.01 to 0.12)	0.03 (−0.02 to 0.09)	0.251
ΔFEV_1_ (9M–12M)	−0.09 (−0.17 to 0.02)	−0.09 (−0.24 to 0.11)	−0.09 (−0.15 to −0.01)	0.961
ΔFEV_1_ (baseline–12M)	−0.04 (−0.14 to 0.09)	−0.02 (−0.14 to 0.13)	−0.05 (−0.14 to 0.09)	0.592

Data are presented for FEV_1_ (liters) at “baseline,” 9M, and 12M and the change (ΔFEV1) for intervals (baseline–9M), (9M–12M), and (baseline–12M). Median (25%–75% interquartile range), Mann-Whitney U test for unpaired cohort comparisons of “low risk” versus “high-risk” cohorts (*P* < 0.05).

ΔFEV_1_, change in forced expiratory volume-1 s; dd-cfDNA, donor-derived cell-free DNA; IQR, interquartile range; TBBx, transbronchial biopsy.

**FIGURE 5.**
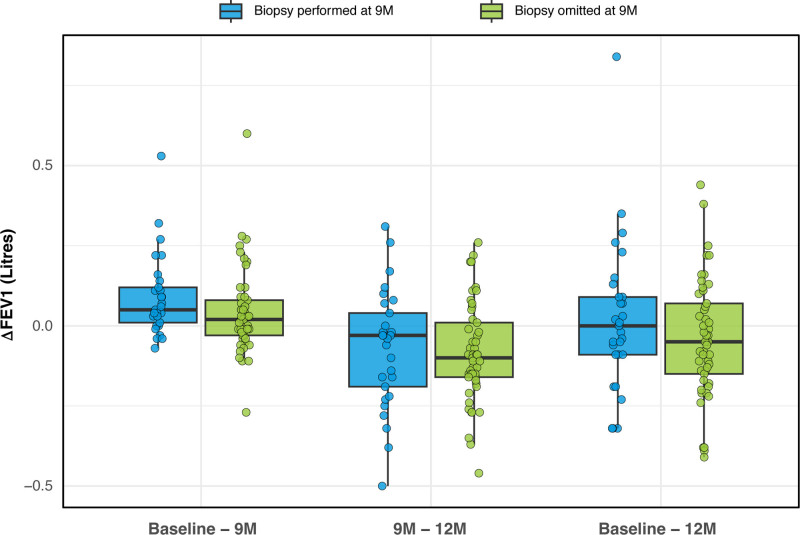
ΔFEV_1_ (liters) for “baseline” to 9M, 9M to 12M, and baseline to 12M timepoints for the “performed” or “omitted” 9M biopsy cohorts. Box and Whisker Plot – median (line), box (25%–75% IQR), Whiskers (1.5 × IQR), outlier points. ΔFEV_1_, change in forced expiratory volume-1 s; IQR, interquartile range.

A similar comparison was performed focusing on the biopsy-omitted and biopsy-performed performed cohorts; again, no significant differences were observed (Table 2; Figure 5). For the 9M cohort where the TBBx procedure had been performed regardless of an associated “low-risk” dd-cfDNA result (ie, “clinically indicated”; 24% [13/54] patients), only one patient demonstrated persistent ACR (ISHLT grade A1Bx) at the 9M and 12M protocol TBBx timepoints; this patient had experienced an episode of ISHLT grade A1Bx rejection on an earlier (6M) protocol TBBx. For the “clinically indicated” 9M TBBx cohort (N = 13), the ΔFEV_1_ (baseline–12M) was 0.03 (−0.06, 0.13) L. There was no difference between the omitted versus performed cohorts (*P* = 0.271) or between the “low-risk” versus “high-risk” dd-cfDNA cohorts (*P* = 0.592) for the ΔFEV_1_ baseline–12M interval.

**TABLE 2.** Data stratification by TBBx that was either “performed” or “omitted” at 9M timepoint

	Overall, N = 78	“Performed” TBBx, N = 29	“Omitted” TBBx, N = 49	*P*
dd-cfDNA (%)	0.56 (0.24–1.09)	1.47 (0.27–2.38)	0.49 (0.16–0.73)	<0.0001
FEV_1_ baseline (L)	2.04 (1.81–2.60)	2.01 (1.79–2.54)	2.05 (1.85–2.66)	0.598
FEV_1_ at 9M	2.06 (1.68–2.51)	2.05 (1.59–2.46)	2.06 (1.76–2.51)	0.505
FEV_1_ at 12M	2.12 (1.83–2.62)	2.05 (1.70–2.62)	2.14 (1.86–2.59)	0.438
ΔFEV_1_ (baseline–9M)	0.04 (−0.01 to 0.11)	0.05 (0.01–0.12)	0.02 (−0.03 to 0.08)	0.052
ΔFEV_1_ (9M–12M)	−0.09 (−0.17 to 0.02)	−0.03 (−0.19 to 0.04)	−0.10 (−0.16 to 0.01)	0.620
ΔFEV_1_ (baseline–12M)	−0.04 (−0.14 to 0.09)	0.00 (−0.09 to 0.09)	−0.05 (−0.15 to 0.07)	0.271

Data are presented for forced expiratory volume-1 second (FEV_1_) (Liters) at “baseline,” 9M, and 12M and the change (ΔFEV1) for intervals (baseline-9M), (9M-12M), and (baseline-12M). Median (25%-75% interquartile range), Mann-Whitney U test for unpaired cohort comparisons of performed TBBx versus omitted TBBx cohorts (p < 0.05).

ΔFEV_1_, change in forced expiratory volume-1 s; dd-cfDNA, donor-derived cell-free DNA; IQR, interquartile range; TBBx, transbronchial biopsy.

### There was no Difference in Development of De Novo HLA Class I or II DSA for the Omitted 9M Protocol Biopsy Cohort

The incidence of HLA class I or class II DSAs for the interval of 9M to 12M in the study was 2 of 78 patients (2.5%), which were both associated with an elevation in dd-cfDNA fractions (2.38% and 1.59%, respectively) at the 9M timepoint but neither was associated with ACR or antibody-mediated rejection (AMR) at the 9M or 12M timepoints (ie, all TBBx were ISHLT grade A0Bx).

### A Spectrum of Clinical Conditions was Observed in Association With an Elevation in dd-cfDNA Fraction

Clinical review for potential attributable conditions associated with elevation in dd-cfDNA at the 9M timepoint included gastroesophageal reflux and bile aspiration (N = 6), respiratory infection (N = 5), renal dysfunction (N = 1), diastolic cardiac failure (N = 1), ACR (N = 3), suboptimal levels of immunosuppression (N = 4), and metastatic squamous cell skin carcinoma (N = 1).

## DISCUSSION

Noninvasive surveillance for AR or other immunologic complications that affect the lung allograft has been extensively investigated for many decades by interrogating specimens of blood, broncho-alveolar lavage fluid, and exhaled breath. These investigations have targeted myriad mediators related to inflammation and innate and adaptive immunity, including exosomes, proteomics, and volatile organic compounds.^14–21^ However, to date, only dd-cfDNA, representing a proxy for allograft cellular injury, has been validated for AR monitoring after LT with commercial assay availability in the United States.^6-8,22^ Real-world data leveraging dd-cfDNA surveillance and reduction in scheduled protocol TBBx procedures should represent a significant advancement to the transplant field. Analogous to the challenges observed in patient care during the SARS-Co(V)-2 pandemic, this prospective QAPI study attempted to address real-world procedural logistics at our LT center.^23^ Increased patient volume has presented challenges to maintaining a strict TBBx protocol schedule at our transplant center.

There were several principal findings from our QAPI study. First, in our experience, incorporating surveillance dd-cfDNA testing into the decision making process, the 9M protocol TBBx procedure was eliminated in >75% of patients with a “low-risk” dd-cfDNA test result, and the lack of detectable difference in spirometry suggests no adverse impact on allograft function at 12 mo. Representing real-world experiences and observed differences in healthcare provider decision-making, approximately one-quarter of the “low risk” dd-cfDNA results nevertheless resulted in performance of an invasive biopsy at 9M, of which only a minority (2 of 13) had evidence of ACR and was classified as “minimal” (grade A1). Second, although only 8.3% of “high risk” dd-cfDNA results were associated with AR at 9M TBBx histopathology review, an elevation in this biomarker may indicate the presence of other clinically relevant disease states or processes. For example, multiple studies have established that elevated plasma dd-cfDNA fraction may be associated with various types of allograft injury, such as infection and occult bile-acid aspiration pneumonitis^6,22,24,25^—findings that were similarly observed in this study. Additionally, an elevation in dd-cfDNA can predate development of clinically evident AMR by several months.^26^ Furthermore, “molecular AMR” by dd-cfDNA assessment, has predicted the subsequent development of chronic lung allograft dysfunction with a hazard ratio of 2.0.^27^ Finally, replacement of the 9M protocol TBBx with dd-cfDNA testing would be expected to reduce the rate of procedure-related serious adverse^28^ complications. Although such complications are acknowledged risks of TBBx procedures, they may be less well-tolerated among frail or single-lung transplanted patients.

Any discussion of noninvasive biomarker surveillance would not be complete in the current healthcare environment without consideration of costs and economics. According to 2021–2022 Medicare claims data, $3971 in costs were directly associated with an outpatient transbronchial biopsy.^29^ When costs related to complications were also factored into this estimate^30^ and adjusted by likelihood,^31–34^ the figure increased to $4442 (**Supplement Table 1, SDC**, https://links.lww.com/TXD/A821). Private insurance costs for an outpatient transbronchial biopsy were projected to exceed $10 000.^35^ Actual charges for dd-cfDNA assays vary significantly based on differences in reimbursement between Medicare and private payors, but are universally lower than these TBBx costs, with an upper limit of $2753.^36^ These numbers clearly demonstrate the potential cost savings associated with implementation of noninvasive plasma dd-cfDNA surveillance and the associated decrease in invasive protocol biopsy procedures.

Limitations to our study are several and are principally related to the inherent challenges in sample size and the lack of histopathological diagnosis at the 9M timepoint. However, this QAPI design intentionally adopted a protocol recommending the inclusion of an SOP TBBx at 12 mo for all patients. This strategy was developed to mitigate any potential risk associated with an undetected episode of asymptomatic “subclinical” ACR. Furthermore, the observed stability in spirometry measurements without evidence for decline from baseline to 12 mo provided additional clinical corroboration against an increased incidence of subclinical rejection in this cohort.

This QAPI study suggested that, when combined with other SOP assessments, surveillance of dd-cfDNA% can help direct the safe omission of the 9M protocol TBBx in >75% of low-risk by dd-cfDNA patients during the first year post-lung transplant and without discernible differences in allograft physiological function, de novo DSA, or later increased the risk of ACR. Further studies focused on strategic omission of protocol TBBx procedures in a multicenter context are needed to corroborate and extend these results. In conclusion, the clinical utility of a combined surveillance framework, assessing both plasma dd-cfDNA and remote daily spirometry metrics, should be a fruitful area of clinical endeavor in providing a precision medicine approach to LT patient care.

## Supplementary Material


