# Supplementary material for: Surveillance Donor-derived Cell-free DNA Allows for the Safe Reduction in Protocol Transbronchial Biopsies After Lung Transplantation
Source: Transplant Direct. 2026 Jan 12;12(2):e1901. doi: 10.1097/TXD.0000000000001901 (PMC12794981; doi:10.1097/TXD.0000000000001901)
Supplement: Supplementary file 1 [file txd-12-e1901-s001.pdf]

SUPPLEMENT for Goyal, et al., “Surveillance Donor-derived Cell-free DNA Allows for the Safe Reduction in Protocol Transbronchial Biopsies after Lung Transplantation”

Health Economic Analysis using Medicare cost estimates.

Procedure	Cost
Transbronchial biopsy, outpatient[a] (CPT code: 31628)<br>Hospital component:<br>Physician component:<br>Histopathology costs:	 $3,568<br>$171<br>$232
Treatment of intermediate complications resulting from a transbronchial biopsy; private insurance.[b]<br>Estimated Medicare costs for intermediate complications resulting from a transbronchial biopsy, based on adjusting private insurance costs downwards by 1/2.54[c]	$18,985<br>$7,474
Average cost of complications per biopsy, assuming 6.35% complication rate[d-g] (the lowest rate of those reported in literature)	$471
Estimated total average cost of a transbronchial biopsy	$4,442

[a] Centers for Medicare & Medicaid Services. Medicare 5% physician/supplier Part B claims limited data set (LDS) standard analytic file (SAF), calendar years 2021–2022. U.S. Department of Health and Human Services. Available from https://resdac.org

[b] Huo J, Xu Y, Sheu T, Volk RJ, Shih Y-CT. Complication Rates and Downstream Medical Costs Associated With Invasive Diagnostic Procedures for Lung Abnormalities in the Community Setting. JAMA Internal Medicine. 2019;179(3):324-332.

[c] RAND Corporation. (2024, May 13). Private health plans during 2022 paid hospitals 254 percent of what Medicare would pay. https://www.rand.org/news/press/2024/05/13.html

[d] Hopkins PM, Aboyoun CL, Chhajed PN, et al. Prospective analysis of 1,235 transbronchial lung biopsies in lung transplant recipients. J Heart Lung Transplant. 2002;21(10):1062-1067.

[e] Trulock EP, Ettinger NA, Brunt EM, Pasque MK, Kaiser LR, Cooper JD. The role of transbronchial lung biopsy in the treatment of lung transplant recipients. An analysis of 200 consecutive procedures. Chest. 1992;102(4):1049-1054.

[f] De Hoyos A, Chamberlain D, Schvartzman R, et al. Prospective assessment of a standardized pathologic grading system for acute rejection in lung transplantation. Chest. 1993;103(6):1813-1818.

[g] Baz MA, Layish DT, Govert JA, et al. Diagnostic yield of bronchoscopies after isolated lung transplantation. Chest. 1996;110(1):84-88.
